# An inducible ectopic expression system of EWSR1-FLI1 as a tool for understanding Ewing sarcoma oncogenesis

**DOI:** 10.1371/journal.pone.0234243

**Published:** 2020-06-05

**Authors:** Daniel J. García-Domínguez, Lourdes Hontecillas-Prieto, Eduardo Andrés León, Sara Sánchez-Molina, Pablo Rodríguez-Núñez, Francisco J. Morón, Nabil Hajji, Carlos Mackintosh, Enrique de Álava

**Affiliations:** 1 Institute of Biomedicine of Seville (IBiS), Hospital Universitario Virgen del Rocío/CSIC/Universidad de Sevilla/CIBERONC, Seville, Spain; 2 Bioinformatics Unit, Instituto de Parasitología y Biomedicina “López-Neyra”, Consejo Superior de Investigaciones Científicas (IPBLN-CSIC), Granada, Spain; 3 Developmental Tumor Biology Laboratory, Institut de Recerca Pediàtrica—Hospital Sant Joan de Déu, Esplugues de Llobregat, Barcelona, Spain; 4 Genomics Core Facility, Instituto de Biomedicina de Sevilla (IBiS), Hospital Universitario Virgen del Rocío/Consejo Superior de Investigaciones Científicas/Universidad de Sevilla, Sevilla, Spain; 5 Division of Brain Sciences, The John Fulcher Molecular Neuro-Oncology Laboratory, Imperial College London, London, England, United Kingdom; 6 Vitro SA, Granada, Spain; 7 Department of Normal and Pathological Cytology and Histology, School of Medicine, University of Seville, Seville, Spain; Hirosaki University Graduate School of Medicine, JAPAN

## Abstract

The presence of the chimeric EWSR1-FLI1 oncoprotein is the main and initiating event defining Ewing sarcoma (ES). The dysregulation of epigenomic and proteomic homeostasis induced by the oncoprotein contributes to a wide variety of events involved in oncogenesis and tumor progression. Attempts at studying the effects of EWSR1-FLI1 in non-tumor cells to understand the mechanisms underlying sarcomagenesis have been unsuccessful to date, as ectopic expression of EWSR1-FLI1 blocks cell cycle progression and induces apoptosis in the tested cell lines. Therefore, it is essential to find a permissive cell type for EWSR1-FLI1 expression that allows its endogenous molecular functions to be studied. Here we have demonstrated that HeLa cell lines are permissive to EWSR1-FLI1 ectopic expression, and that our model substantially recapitulates the endogenous activity of the EWSR1-FLI1 fusion protein. This model could contribute to better understanding ES sarcomagenesis by helping to understand the molecular mechanisms induced by the EWSR1-FLI1 oncoprotein.

## Introduction

Ewing sarcoma (ES) is the second most frequent sarcoma of bone and soft tissues in children and young adults. ES is characterized by various fusions involving the EWSR1 and ETS transcription factors, with EWSR1-FLI1 the most common [1, 2]. One of the burning questions in ES is whether the translocation is the initiating event. Recently, Anderson et al. have demonstrated that the first event in the ES oncogenesis is the chromosomal translocation, resulting in its fusion protein product EWS-ETS [3]. Accordingly, the EWS-ETS fusion protein is also important for ES progression and oncogenic potential [4–7]. The sustained expression of this fusion protein allows cells to acquire oncogenic features that trigger multiple genetic and epigenetic modifications [8–11], among other events. To understand the role of the EWSR1-FLI1 protein in the development of ES, we first need to model its induction in the cell of origin; however, this is a challenge as the cell origin of ES is still unknown. For that reason, researchers commonly take one of two strategies: i) use EWSR1-FLI1 knockdown in ES cell lines, or ii) use a heterologous (non-ES related) system that expresses EWSR1-FLI1. Unfortunately, however, inhibiting EWSR1-FLI1 in ES cell lines induces apoptosis [7, 12], and ectopic expression of EWSR1-FLI1 prompts apoptosis and growth arrest in mouse normal embryonic fibroblasts as well as in primary human fibroblasts [13, 14]. Moreover, ectopic EWSR1-FLI1 expression in human mesenchymal stem cells (hMSCs), defined as the putative cells of origin for ES, does not result in cell transformation or in tumor formation in immunocompromised mice, although hMSCs are initially permissive to EWSR1-FLI1 ectopic expression [15, 16]. Hence, having a suitable *in vitro* model that ectopically expresses EWSR1-FLI1 is an essential preliminary requirement for researchers to begin to understand the molecular mechanism induced by EWSR1-FLI1 emergence.

To construct a suitable heterologous system, it is essential to achieve a sustained expression of the fusion protein without compromising either cellular functions or cell viability. In addition, integration of a FLAG-tag element into the system would be useful to allow a more sensitive detection of DNA/protein or protein/protein interactions [17] when antibodies are not efficient or specific enough to get a high signal-to-noise ratio. The use of the FLAG-tag strategy to immunoprecipitate EWSR1-FLI1 has been extensively reported [7, 9, 18].

In this study, our goal is to develop an inducible ectopic EWSR1-FLI1 system that can be used to get additional insights into the mechanisms by which EWSR1-FLI1 modulates cell transformation and drives ES tumorigenesis and aggressiveness. To this aim, we used a permissive HeLa cell line, thereby avoiding the problems due to the ectopic expression of EWSR1-FLI1 and enabling us to study it also long-term. Further, we included a C-terminal 3×FLAG tag and verified that it did not disrupt the activity of the fusion protein. Altogether, our ectopic and inducible EWSR1-FLI1 3×FLAG model circumvents the problems of apoptosis and cell cycle interference. Thus, it could provide a useful tool for studying the molecular mechanisms induced by the emergence and maintenance of EWSR1-FLI1.

## Materials and methods

### Cell lines and cell culture conditions

The HeLa Tet-On^®^ 3G Cell Line (Clontech; 631183), which expresses the tetracycline (Tet)-regulated transactivator Tet-On 3G, was established as a parental cell line. Cells were grown in DMEM with 10% FBS, and 250 μg/ml G418 (Gibco; 11811031) was added to maintain the transfected vector. The stable EWSR1-FLI1 HeLa clones grew in the same medium but supplemented with 0.5 μg/ml of puromycin (SIGMA; A1113803). Both antibiotics were removed from the medium culture prior to carrying out experiments. Doxycycline (Clontech; 631311) was used to induce the system. The ES cell lines A673 and RDES were obtained from ATCC (#CRL-1598 Lot 58078570 and #HTB-166 Lot 58078725, respectively). Cells were grown on 0.1% gelatin-coated plates in DMEM 10% FBS (A673) and RPMI 15% FBS (RDES). All cell lines were maintained in 37°C incubators, in an atmosphere of 5% CO_2_. Cell lines were free of mycoplasma, and were continuously screened with the MycoAlert^®^ Mycoplasma Detection Kit (Lonza) to ensure this.

### Establishment of HeLa cell lines expressing inducible EWSR1-FLI1 (7–6) 3×FLAG C-terminal

The HeLa Tet-On^®^ 3G cell line (Clontech, #631183) was used to achieve inducible and regulated expression of EWSR1-FLI1. This cell line constitutively expresses the reverse tetracycline-responsive transcriptional activator characterized by high sensitivity to doxycycline (Clontech, #631310). For this, the EWSR1-FLI1 (7–6 type) cDNA (in which the original stop codon was replaced for a triple FLAG-stop codon sequence) was first cloned into the response vector pRetroX-Tight-Pur (Clontech, #632104). After retroviral infection, cells were selected using 250 ゼg/ml G418 (Gibco, #11811–031) and 0.6 ゼg/ml puromycin (SIGMA, #18833), to obtain stably-transfected individual clones that conserved both plasmids. Finally, individual clones were selected that had no leakage and were capable of expressing ectopic EWSR1-FLI1 upon doxycycline induction. EWSR1-FLI1 expression was analyzed by Western blot using an antibody against the COOH-terminal region of FLI1, which was present in this chimeric protein (clone C-19) (Santa Cruz, #SC-356).

### Protein extraction and Western blot

Protein extracts in RIPA buffer (150 mM NaCl, 1% (v/v) NP40, 50 mM Tris-HCl pH 8.0, 0.1% (v/v) SDS, 1 mM EDTA and 0.5% (w/v) deoxycholate) supplemented with protease inhibitor, 10 mM NaF and 2 mM NaOv, were resolved in 8%/12% polyacrylamide gels (40 μg of protein per lane) and transferred to a PVDF membrane (Bio-Rad). Primary antibodies were incubated overnight at 4°C (1:200 to 1:1000 dilution), and EWSR1-FLI1 expression was determined using the anti-FLI1 antibody (C-19) (Santa Cruz, #SC-356), calnexin (E-10) (Santa Cruz, #SC-46669), FLAG-M2 (SIGMA, #F3165) and LSD1 (Cell Signaling, #C69G12).

### Proliferation and cell viability assay

Cells were seeded in 96-well culture plates with 2000 cells per well and cultured for 24 h, 48 h and 72 h (parental Hela3G or the #3.10 and #3.15 selected clones). Doxycycline was added to complete growth medium at 1 μg/ml concentration. Cell viability for the indicated times was determined using the ATPlite kit (PerkinElmer, Waltham, MA, USA), and inhibitory concentrations of proliferation were calculated. Relative units obtained were normalized with respect to the 0 h control point.

### mRNA isolated and qRT-PCR

HeLa cells RNA was isolated using miRVana miRNA Isolation Kit (Ambion; Life Technologies, USA). A Nanodrop ND-2000 Spectrophotometer (Thermo Scientific) was used to evaluate the quantity and quality of the total RNA. Expression of selected genes was analyzed by qRT-PCR as described in García-Domínguez et al. [19]. TaqMan probes used in this study are listed in S1 Table.

### Chromatin immunoprecipitation (ChIP)

HeLa cells were fixed with DSG [Di(N-succinimidyl) glutarate] 0.2 mM for 45 min at room temperature followed by 1% formaldehyde at room temperature for 10 min, and the washed once in ice-cold PBS. The pellet was resuspended in lysis buffer (0.1% SDS, 0.1 M NaCl, 1% Triton X-100, 1 mM EDTA, 20 mM Tris pH 8 and 1 mg/ml protease inhibitors) and sonicated with a Bioruptor until the crosslinked chromatin was sheared with an average DNA fragment length of 0.5 kbp. After centrifugation (30 min at 14000 rpm), chromatin preparations were precleared by incubation with 40 μl of Protein A agarose/salmon Sperm DNA 50% gel slurry (Millipore) for 2 h at 4°C under rotation. Protein A agarose was removed by centrifugation, and the precleared chromatin was immunoprecipitated by incubation with 5 μg anti-FLAG-M2 antibody and 50 μl protein A agarose overnight at 4°C. Washed pellets were eluted with 120 μl of a solution containing 1% SDS, 0.1 M NaHCO_3_. Eluted pellets were de-crosslinked at 65°C overnight and purified in 50 μl Tris-EDTA buffer using the QIAquick PCR Purification Kit (Qiagen). Differences in the DNA content from every immunoprecipitation assay were determined by real-time PCR using the ABI 7700 sequence detection system and SYBR Green master mix protocol (Applied Biosystems). Primers used in this study are listed in S1 Table. The reported data represent real-time PCR values normalized to input DNA and expressed as percentage (%) of bound/input signal.

### Co-immunoprecipitation (co-IP)

Whole protein extracts (250 ゼg) in NP40 buffer (150 mM NaCl, 20 mM Tris pH 8.0, 1 mM DTT, 0.5% NP40) were incubated with 15 ゼl of protein A dynabeads (Invitrogen) coupled to 2 ゼg of LSD1 antibody (Cell Signaling) for 3 h at 4°C. After magnetic immunoprecipitation and washes, immunoprecipitates were resolved in a 10% polyacrylamide SDS-PAGE gel, transferred and analyzed by Western blot as described above.

### Gene‐chip hybridization and transcriptome analysis

Microarrays analyses were conducted using GeneChip^®^ Human Transcriptome 2.0 Array (Thermo Fisher Scientific, Inc.) with the GeneChip^®^ technology microarrays, including a Fluidics Station 450 and a Scanner 3000 (Thermo Fisher Scientific, Inc.). Following the procedures described in the user manual, the amplification and reverse transcription of 100 ng of total RNA was performed for three biological replicates of HeLa cells. Amplified cDNA (5.5 ゼg) was then fragmented and labeled for hybridization to the cartridge arrays and subsequent scanning. Gene‐level differential expression analysis was performed using CEL files that contain the original values produced by the scanner. CEL files were analyzed by Transcriptome Analysis Console (TAC) 4.0 software (Thermo Fisher Scientific, Inc.) for statistical analysis, providing a list of differentially expressed genes. Previously published data, including Riggi et al. [16] (hMSC_EWSR1-FLI1) and GSE14543 (ES cell lines shEWSR1-FLI1), were compared to our results in the HeLa model.

### GSEA preranked calculation

Gene Set Enrichment Analysis (GSEA) from the Broad institute [20] was used to interpret the resulting expression profile from this work and to find the most similar signature in the Molecular Signatures Database (MSIgDB) [21]. This database is a collection of annotated gene sets to be used with GSEA to help represent a wider range of biological processes and diseases from transcriptomic analyses.

In this study, all differentially expressed genes (p-value < 0.05 and logfc>|1.3|) were considered and sorted based on their fold-change to perform a GSEA preranked analysis. This algorithm highlights genes that have a greater impact as compared to all genes with altered expression, and identifies other gene sets from MSigDB comprising a set of genes, similar to those highlighted by GSEA. The greater the similarity, the greater the enrichment value (defined as normalized enrichment score [NES]) between both types of data. The set of differentially expressed genes obtained in this study faced the curated dataset of MSigDB, called c2, which is represented by databases such as KEGG, REACTOME, Gene Ontology (GO) and data published by other authors with experimental evidence; the c2 dataset comprises 5501 gene sets.

### Statistical analyses

Graphpad Prism software, version 6.01, was used for statistical analyses. The statistical tests used are detailed in the corresponding Result sections. 2way ANOVA using the Sidak´s multiple comparison was used to compare proliferation rates in the present or absence of doxycycline. To compare the two groups, multiple t-test was used. Data are presented as mean ± SEM. For all analyses, p-values of ≤0.05 were considered statistically significant. All experiments were carried out in triplicate.

## Results

### The HeLa3G cell line is permissive to EWSR1-FLI1 ectopic expression

**Fig 1:**
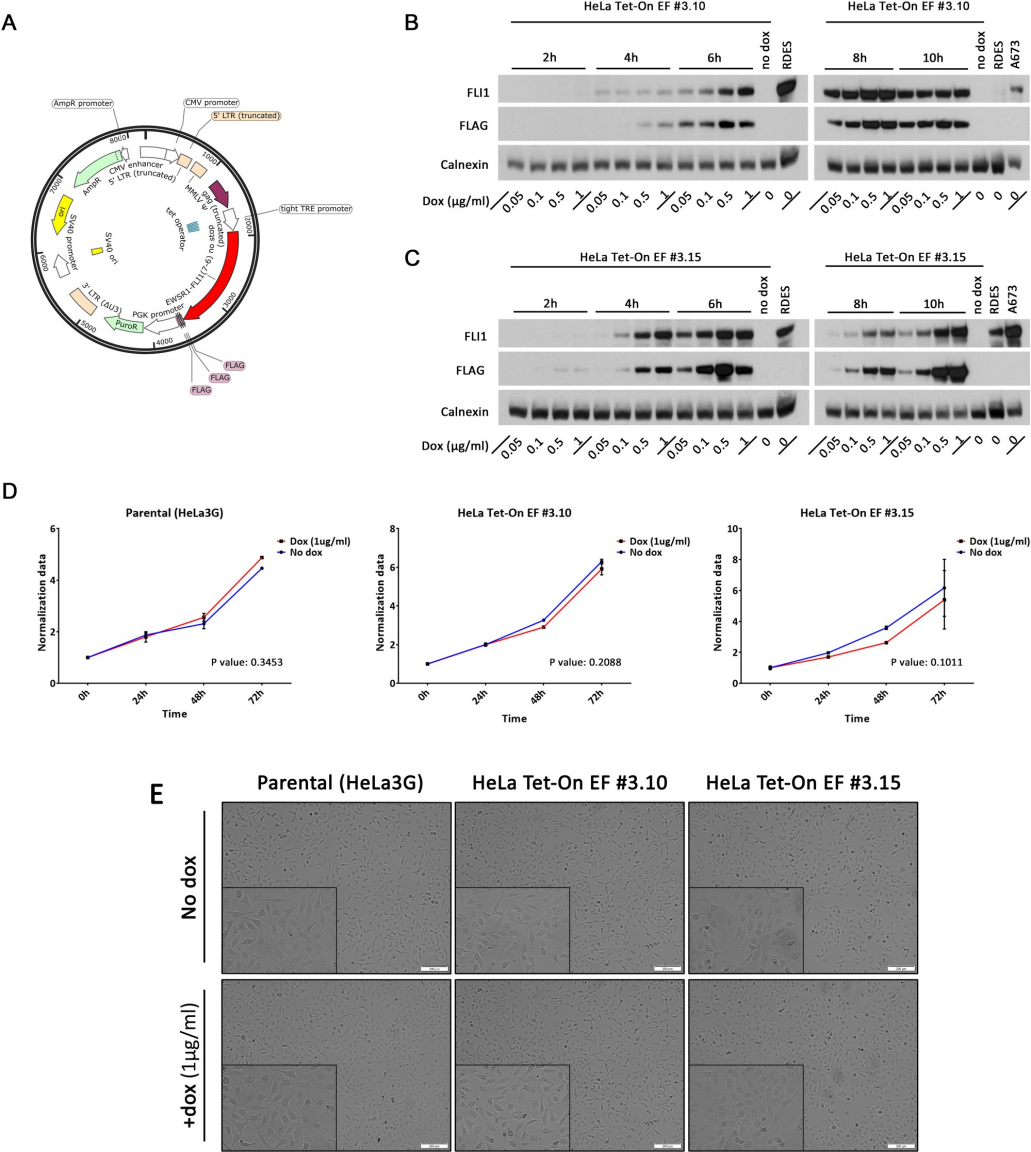
EWSR1-FLI1 expression in a HeLa Tet-On model. **A.** Schematic diagram of the retroviral vector used in the study. **B, C.** Western blot analysis showing the inducible expression of EWSR1-FLI1 in two HeLa Tet-On EF clones (#3.10 and #3.15) using the FLI1 and FLAG antibodies in the presence of different concentrations of doxycycline at different time points. The RDES and A673 ES cell lines were used as controls for FLI expression. **D.** Proliferation of parental HeLa3G cells (control) and HeLa Tet-On EF #3.10 and #3.15 clones in the presence or absence of doxycycline. Values are mean ± s.d. of three biological independent replicates. Statistical tests: 2-way ANOVA multiple comparisons. **E.** Doxycycline does not affect the morphology of parental cells or the HeLa Tet-On EWSR1-FLI1 models (#3.10 and #3.15). The cell lines were grown in medium with or (for controls) without doxycycline (1 μg/ml) for 18 days. Phase contrast shows 20× and 40× magnifications.

Despite the underlying association between ES and the EWSR1-FLI1 oncogene, ‘*de novo*’ molecular mechanisms induced by the fusion protein are not fully understood. To develop a model that could help to elucidate these, a retroviral vector (EWSR1-FLI1 3×FLAG C-terminal) was designed to generate HeLa cells that express EWSR1-FLI1 under the control of a doxycycline-inducible promoter (Fig 1A). Stably transfected HeLa Tet-On cells encoding the EWSR1-FLI1 (HeLa Tet-On EF) were made from the parental HeLa3G cells (control) and a vector that constitutively expresses the tetracycline-controlled transactivator. Single clones with only one plasmid copy and with no leakage of the system were previously preselected.

To characterize its induction levels, EWSR1-FLI1 was induced at different time points in HeLa Tet-On EF #3.10 and #3.15 clones, and at different concentrations of doxycycline. The results showed a dose- and time-dependent increase of EWSR1-FLI1 induction. The RDES and A673 ES cell lines were used as a positive control, and clones without doxycycline were used as a negative control for the induction system (Fig 1B and 1C). We observed an analogous EWSR1-FLI1 protein level in both clones with respect to the ES cell lines.

We next evaluated the possible effects of the ectopic fusion protein expression and the doxycycline on cell growth in HeLa cells in a proliferation assay. No changes in proliferation rates were observed in parental HeLa3G cells at different time points, hence the doxycycline effect was disregarded (Fig 1D). Moreover, no significant alterations in cell growth were observed in the first 72 h in either of the HeLa Tet-On EF clones (Fig 1D) (although the #3.15 clone showed a slight, not significant proliferation reduction when the EWSR1-FLI1 was induced; Fig 1D). Long-time doxycycline induction (18 days) did not trigger morphologic changes in the parental or transformed HeLa clones (Fig 1E).

Altogether, we obtained an ectopic and inducible EWSR1-FLI1 3×FLAG C-terminal system in a permissive HeLa cell line. The selected clones can express ‘*de novo*’ the EWSR1-FLI1 fusion protein without inducing modifications in their proliferation over a short or long term, and without compromising cell viability.

### Gene expression patterns of ectopic EWSR1-FLI1 in HeLa and endogenous EWSR1-FLI1 are similar

**Fig 2:**
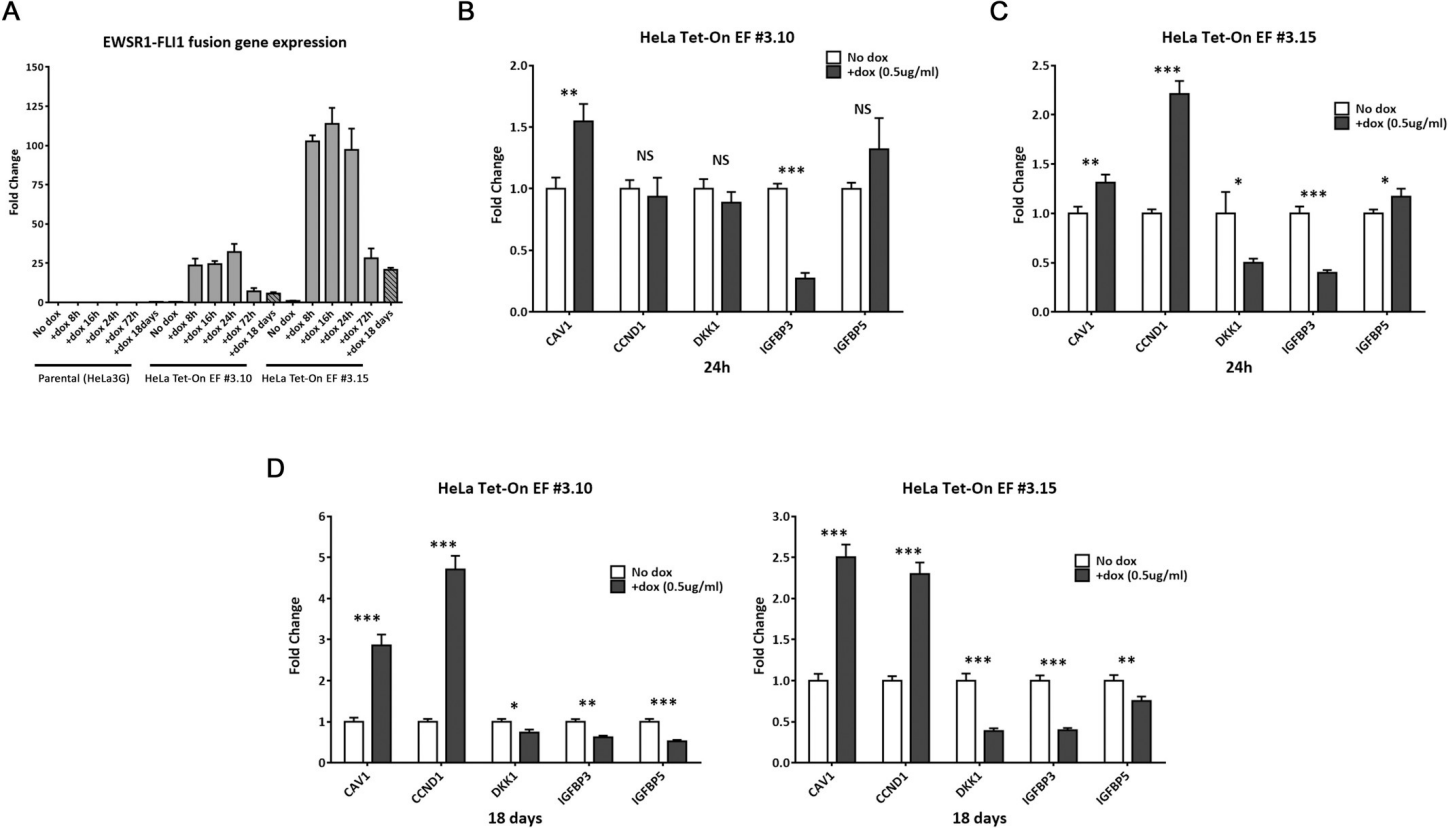
The HeLa model expresses the aberrant transcription factor EWSR1-FLI1. **A.** EWSR1-FLI1 gene fusion expression analysis in parental HeLa3G cells (control) and two different clones (HeLa Tet-On EF #3.10 and #3.15) in the presence or absence of doxycycline at different time points. **B–D.** EWSR1-FLI1 up- and downregulated target genes are shown after 24 h and 18 days of doxycycline induction. Values are given as mean ± s.d. of three biological independent replicates. Statistical tests: significant analysis of multiple t-test; ***, <0.001; **, 0.01;*, 0.05; NS, not significant.

EWSR1-FLI1 is widely described to act as an aberrant transcription factor that regulates the expression of multiple target genes [5, 7, 22]. To test the HeLa system expressing the chimeric protein, we first analyzed the expression of EWSR1-FLI1 by RT-qPCR in the parental control (HeLa3G) and the two different clones in the presence or absence of doxycycline at different time points. Notably, aberrant transcription factor expression was detected in both clones after doxycycline administration, and there was no EWSR1-FLI1 expression in parental HeLa3G in the presence of doxycycline (Fig 2A). Second, we evaluated some well-known specific targets genes that are regulated by EWSR1-FLI1. For this, the doxycycline impact was corrected by comparing the parental control (HeLa3G) in presence or absence of doxycycline in the gene expression quantifications; these effects were corrected in the HeLa Tet-On EF #3.10 and #3.15 clones. Indeed, we observed increased (CAV1 and CCDN1) or decreased (DKK1, IGFBP3 and IGFBP5) expression of genes up- or downregulated by EWSR1-FLI1, respectively (Fig 2B, 2C and 2D). These results were consistent at different time points, and the fold-change values of expression of these genes increased after 18 days of induction (Fig 2D).

Hence, we have shown that ‘*de novo*’ EWSR1-FLI1 expression in our model acts as an aberrant transcription factor and modulates the expression of common targets genes, analogously to the endogenous EWSR1-FLI1 in ES cell lines. This regulatory effect of EWSR1-FLI1 is sustained over a long period of time.

### Ectopic EWSR1-FLI1 also interacts with specific DNA-binding sites and specific protein complexes

**Fig 3:**
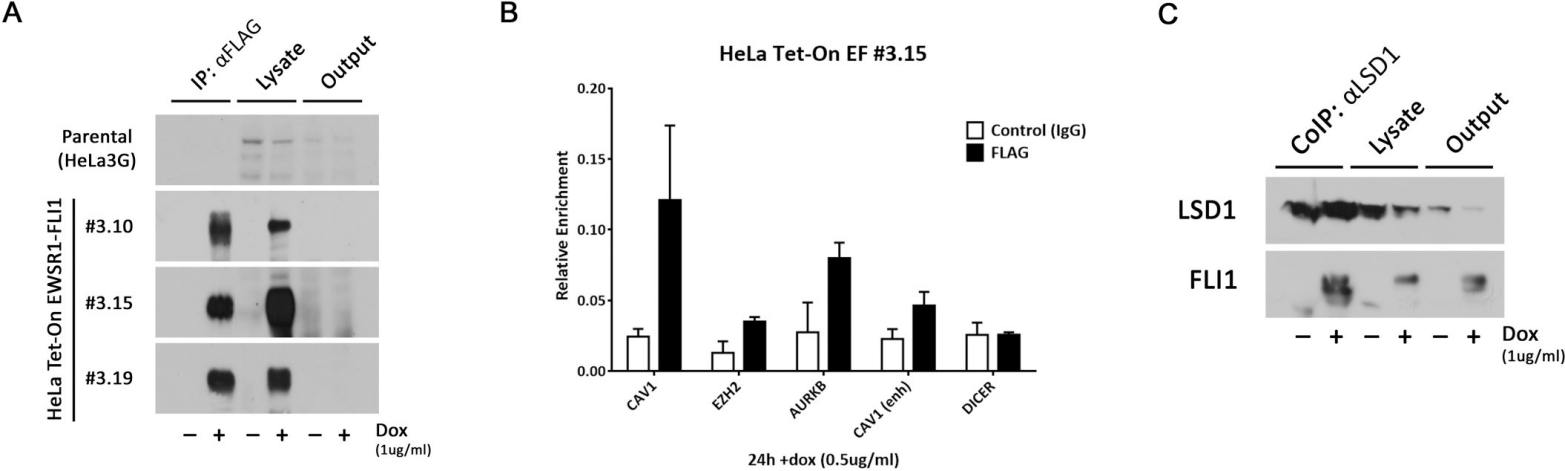
Ectopic EWSR1-FLI1 interacts with specific DNA and protein-binding sites. **A.** EWSR1-FLI1 immunoprecipitation using anti-FLAG antibody in parental HeLa3G cells (control) and two different clones (HeLa Tet-On EF #3.10 and #3.15) in the presence or absence of doxycycline. **B.** Binding of EWSR1-FLI1 to the *CAV1* enhancer and the promoters of the *CAV1*, *EZH2* and *AURKB* genes. qPCR analysis of EWSR1-FLI1 ChIP signals for HeLa Tet-On EF #3.15 clone. **C.** Co-immunoprecipitation of EWSR1-FLI1 3×FLAG C-terminal and LSD1 protein in HeLa Tet-On EF #3.15 clone.

The ability to bind to the promoter regions of its target genes is essential for EWSR1-FLI1’s modulation of gene expression as well as for facilitating protein-protein complexes. To test whether the ectopically expressed EWSR1-FLI1 also has this ability, immunoprecipitation is required. Several studies were able to immunoprecipitate EWSR1-FLI1 using a FLAG-tag strategy approach [7, 9, 18] but not using a FLI1 antibody because of its background noise and low sensitivity. We were likewise unable to immunoprecipitate EWSR1-FLI1 using an HA-Tag at its C-terminus. Therefore, we tested a 3×FLAG at the C-terminus in this HeLa system (Fig 1A). To immunoprecipitate the specific 3×FLAG-tagged EWSR1-FLI1, we tested individual and different HeLa Tet-On EF clones. Immunoblot results showed that EWSR1-FLI1 was immunoprecipitated with an anti-FLAG antibody; as a control, no immunoprecipitation was observed in the absence of doxycycline (Fig 3A). Therefore, we concluded that adding the 3×FLAG to the C-terminus allows the EWSR1-FLI1 in HeLa model to be immunoprecipitated.

We then aimed to validate the ability of the fusion protein to bind to specific DNA promoter and enhancer sequences. We evaluated the binding of EWSR1-FLI1 protein to *CAV1*, *EZH2* and Aurora kinase B (*AURKB*) promoters as well as to the *CAV1* enhancer in HeLa Tet-On EF #3.15 clone. After doxycycline induction, we observed that ectopically expressed EWSR1-FLI1 was bound specifically to the *CAV1*, *EZH2* and *AURKB* promoters and to the *CAV1* enhancer (Fig 3B). Notably, no EWSR1-FLI binding was observed at the *DICER* promoter (Fig 3B), ruling out that the binding we observed was due to non-specific binding.

Finally, to study the ability of the ectopically expressed EWSR1-FLI1 to bind specifically and directly to protein complexes, we tested for its interaction with the LSD1 histone demethylase (a NuRD complex subunit), which has been previously reported [23]. Indeed, we observed that LSD1 co-immunoprecipitated with the ectopic EWSR1-FLI1 in HeLa Tet-On EF #3.15 clone only in presence of doxycycline (Fig 3C). In addition, we observed a fusion protein band in the output, which could be either free ectopic EWSR1-FLI1 or that bound to other complexes (Fig 3C).

Taken together, these data provide evidence that the ectopic EWSR1-FLI1 is functionally active and similar to the endogenous in its site-specific DNA-binding and protein interaction complexes. In addition, it also suggests the usefulness of the 3×FLAG-tagged model.

### The HeLa Tet-On EWSR1-FLI1 model is a representative model of ES gene expression patterns induced by the chimeric protein fusion

**Fig 4:**
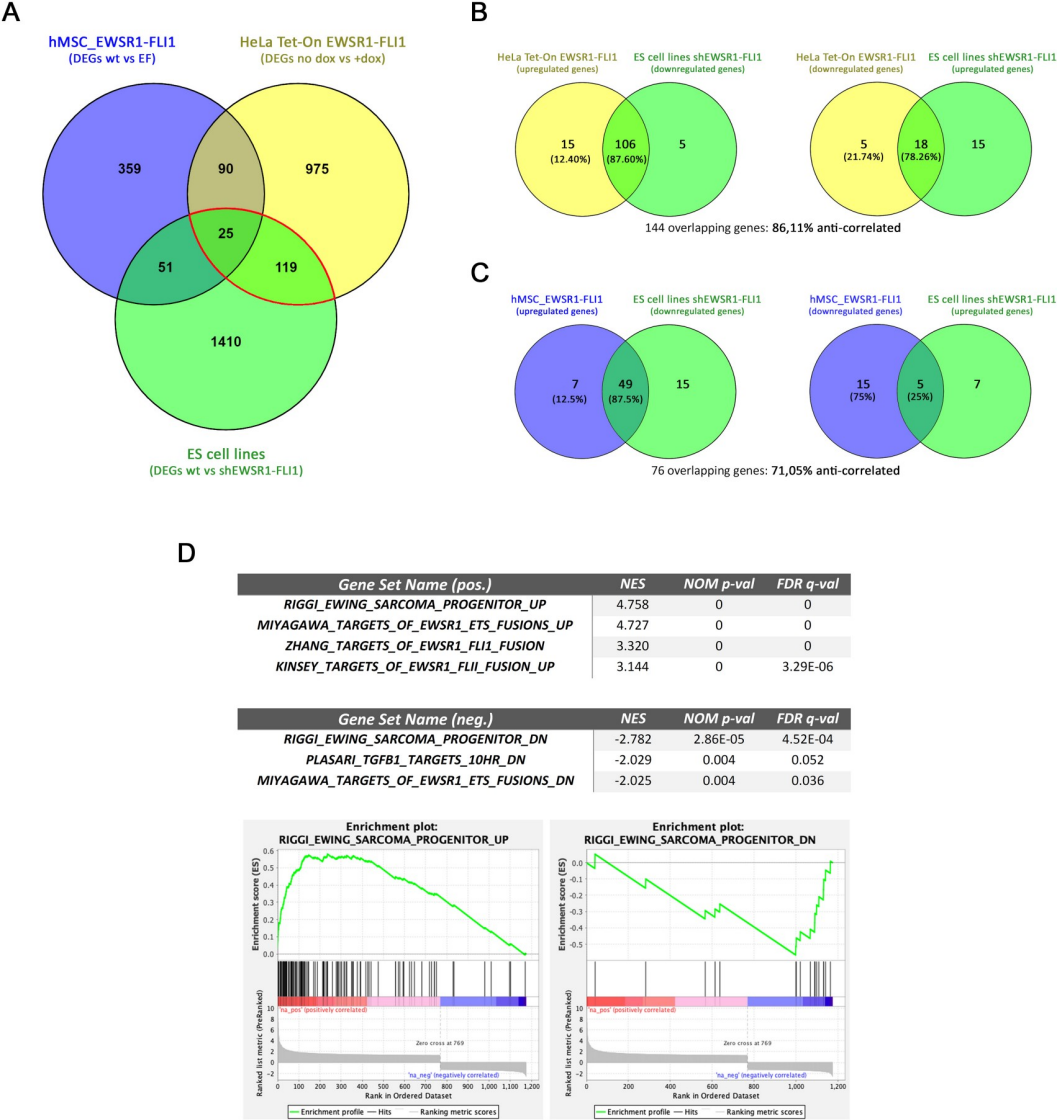
HeLa Tet-On EWSR1-FLI1 gene expression patterns correlated with different published models. **A.** Venn diagram analysis of differentially expressed genes between HeLa Tet-On EWSR1-FLI1, hMSC_EWSR1-FLI1 and the ES cell line shEWSR1-FLI1. **B.** Venn diagrams showing the overlap between upregulated genes in the HeLa model and downregulated genes in ES cell lines shEWSR1-FLI1 (and *vice versa*). **C.** Overlapping genes between hMSC_EWSR1-FLI1 and shEWSR1-FLI1 ES cell line models are shown in analogous Venn diagrams. **D.** MSigDB analysis of genes that were significantly and at least 1.3-fold repressed or induced by EWSR1-FLI1 ectopic expression in HeLa cells shows marked overlap with EWSR1-FLI1 target genes (top). The enrichment plots with the best enrichment score are shown as |NES| (bottom). NES, the normalized enrichment score.

Next, we addressed the ability of the HeLa Tet-On EF cells to modify transcriptome profiling. A transcription gene expression array after 24 h of doxycycline induction revealed a significant modification of 1209 genes in both clones (S2 Table). To determine how representative our HeLa model is with respect to the EWSR1-FLI1 activity in ES, the gene expression array results were compared with previously published data from hMSCs (the putative cell of origin for ES) [16] as well as from five ES cell lines in which the protein fusion was ectopically expressed or depleted by shRNA system [24]. A Venn diagram showed 144 overlapping genes between our HeLa model and the five ES cell lines in which the EWSR1-FLI1 was depletion (the ES cell lines shEWSR1-FLI1), and only 76 common genes between hMSCs that ectopically expressed the fusion protein (hMSC_EWSR1-FLI1) and shEWSR1-FLI1 cell lines (Fig 4A). In addition, analyzing the 144 genes shared by the HeLa model and ES cell lines shEWSR1-FLI1, we confirmed that most upregulated genes in the HeLa model matched with the downregulated genes when EWSR1-FLI1 is depleted in ES cell lines (87.6%) (Fig 4B). Inversely, most genes downregulated in the HeLa model matched with the upregulated genes due to the depletion of EWSR1-FLI1 in ES cell lines (78.26%) (Fig 4B). Nevertheless, the hMSC_EWSR1-FLI1 model shows a lower percentage of anti-correlated genes (71.05%) than shEWSR1-FLI1 ES cell lines (Fig 4C). Accordingly, we consider that our inducible and ectopic system of EWSR1-FLI1 in HeLa cells is a representative model of its action in ES.

To determine whether the overlapping genes were reflective of a similar biological EWSR1-FLI1 transcriptional activity, we expanded our analysis to include all genes that were significantly modulated by ectopic chimeric transcription factor in HeLa cells. After conducting a preranked study with GSEA software, we compared our list of differentially expressed genes (with p<0.05 and fc > |1.3|) against the MS2DB C2 signature database. We observed a statistically significant enrichment in ES signatures (Riggi, Miyagawa, Zhang, Kinsey), with positive normalized enrichment scores (NES) for mostly upregulated genes (UP gene sets), and negative NES for downregulated genes (DN gene sets) in our HeLa model (Fig 4D and S3 Table). GSEA of rank-ordered genes confirmed that the gene signature induced in our model recapitulates the molecular features, and subsequently the biology, of the specific chimeric fusion protein in ES.

## Discussion

In-depth understanding of the molecular mechanisms induced by EWSR1-FLI1 emergence is one of the challenges in the field of ES. So far, many ectopic expression models for the study of fusion protein function have been designed. Unfortunately, depending on the specific cellular model in which EWSR1-FLI1 is expressed, abnormal physiological effects, such as apoptosis, growth arrest [13, 14] or differentiation [25, 26] have hindered the study. To tackle the problem, it is necessary to develop a fusion protein–permissive model, and to avoid that expressed oncogenic fusion proteins trigger functional misbalance.

We have now designed an inducible ectopic EWSR1-FLI1 model to address this challenge. As the cell of origin in ES is unknown, a permissive cell line was used to develop the model [27]. The HeLa cell line showed a strong tolerance to EWSR1-FLI1 after more than 18 days of induction, with no morphological or proliferative alterations. Moreover, the HeLa model was able to express ‘*de novo*’ EWSR1-FLI1 to a similar expression level as that in ES cell lines. It is important to note that there is compelling evidence that preservation of the transcription factor activity of the fusion protein is essential for ES tumorigenesis [4, 5]. Thus, functional studies to confirm this were essential to validate our model. Ectopic EWSR1-FLI1 expression was indeed able to up- and downregulate expression of its specific target genes, analogous to those modified by endogenous EWSR1-FLI1 in ES cells lines [22]. Further, EWSR1-FLI1 had the ability to bind specific promoters, such as in *CAV1* and *EZH2* [28], and to interact with other proteins to allow protein-protein complexes to form, as previously described [23]. Therefore, we conclude that ectopic EWSR1-FLI1 expression in this model reproduces the activity induced by EWSR1-FLI1 endogenous expression.

To study the regulation of gene expression more in detail, we determined the common genes expressed in EWSR1-FLI1–depleted ES cells with respect to our HeLa model and to hMSC cells [16] that ectopically express EWSR1-FLI1. Shared genes were two-fold higher between the EWSR1-FLI1–depleted ES cells and the HeLa model than between EWSR1-FLI1–depleted ES cells and hMSCs. Moreover, genes with increased expression in EWSR1-FLI1–depleted ES cells had a reduced expression in the HeLa model with the EWSR1-FLI1 ectopic expression. Additionally, in an unsupervised manner, we compared the expression signature induced by the EWSR1-FLI1 expression in our HeLa model with the GSEA gene sets (C2_MSigDB). Indeed, our signature correlated significantly with several previously published EWSR1-FLI1 target gene signatures. These data further support the idea that our model recapitulates the molecular features, and therefore the biology, of EWSR1-FLI1 in ES. We conclude from these experiments that the HeLa model provides a representative model of the gene expression patterns induced by the chimeric protein fusion.

In addition to permissiveness and functionality, this model exhibits other important features. For instance, unlike other models with an ectopic, constitutive expression of fusion protein, our inducible model would allow us to assess the initial changes that appear after ‘*de novo*’ EWSR1-FLI1 expression. In addition, our model allows gene expression data to be obtained over short, medium and long terms (whereby “long term” in this study was 18 days post-induction), without compromising cell viability. Overall, the inducible features of the model will allow the identification of specific gene sets that could be altered over different time periods, and well as a comparison between the short- and long-term molecular and physiological events after induction. Likewise, it would useful for studying fluctuations of EWSR1-FLI1 expression levels, and for determining the mechanisms of how these fluctuations are involved in the metastatic process and proliferation [29].

Adding a 3×FLAG-tag to the C-terminal end in this inducible system also represents a technical advantage. Although using a FLI1 antibody is currently the main way used to detect EWSR1-FLI1 in ES, using FLAG-tag strategy in other systems [7, 9, 18] shows that it helps to avoid the background noise and increase detection sensitivity. Indeed, we observed that, in the different HeLa Tet-On EF clones, chimeric protein immunoprecipitation was more effective with the 3×FLAG-tag addition. Thus, we present a solution to difficulties that arise using a FLI1 antibody. For instance, the tag would allow genomics (e.g. ChIP-seq) and proteomics (e.g. mass spectrometry) studies to more precisely detect the EWSR1-FLI1 fusion by increasing the immunoprecipitation sensitivity and background reduction.

Finally, it should be noted that *in vivo* studies are a necessary step in biomedical research. Attempts made to transform non-tumor cells (such as hMSCs, primary pediatric human mesenchymal progenitor cells and human embryonic stem cells with p53 mutated), as these are considered to be potential cells of ES origin, but have been unsuccessful [15, 16, 30]. Although these cells were permissive to EWSR1-FLI1 for at least short times, the ectopic EWSR1-FLI1 expression resulted in neither cell transformation nor tumor formation in mice. However, in our model, ectopic EWSR1-FLI1 expression can be sustained over time, and it can recapitulate the biological features of ES. Results from these experiments could be studied *in vivo*, providing a very useful tool for performing preclinical studies focused on the EWSR1-FLI1 role that cannot be addressed with the previously reported models.

## Conclusions

We have constructed and evaluated an inducible model (Tet-On EWSR1-FLI1 3×FLAG C-terminal) in HeLa cell lines, which ectopically expresses EWSR1-FLI1 and is able to reproduce the endogenous EWSR1-FLI1 function in ES cell lines. This heterologous system (which is non-ES related) addresses an unmet need by providing a suitable and useful tool to study the role of the chimeric fusion protein at the onset of the disease, as well as the alterations induced by the sustained EWSR1-FLI1 expression, which are essential for tumor maintenance.

## Supporting information

S1 Table qRT-PCR Taqman probes and primer used for expression gene validation and ChIP assay respectively. (DOC)

S2 Table Comparative analysis of differentially expressed genes between HeLa Tet-On EWSR1-FLI1 models no activated (no dox) and activated after 24h of doxycycline induction. This table shows only the differentially significant expressed genes (p-value <0.05).
(XLSX)

S3 Table GSEA of rank-ordered genes sets (C2_MSigDB): +dox 24 h HeLa Tet-On EWSR1-FLI1 3×FLAG C-terminal signature. Ranked gene set: NOM p<0.05 and FDR q <0.05.
(PDF)
