# Supplementary material for: An inducible ectopic expression system of EWSR1-FLI1 as a tool for understanding Ewing sarcoma oncogenesis
Source: PLoS One. 2020 Jun 5;15(6):e0234243. doi: 10.1371/journal.pone.0234243 (PMC7274397; doi:10.1371/journal.pone.0234243)
Supplement: S1 Table — (DOC) [file pone.0234243.s001.doc]

| **Supplemental Table 1** | | | | | |
| --- | --- | --- | --- | --- | --- |
| ***qRT-PCR Taqman probes used for gene expression validation*** | | | | | |
| **Genes** | | **Brand** | **Reference** | **Amplicon length** | |
| *CAV1* | | Thermo Fisher | Hs00971716_m1 | 66 bp | |
| *CCND1* | | Thermo Fisher | Hs00765553_m1 | 57 bp | |
| *DKK1* | | Thermo Fisher | Hs00183740_m1 | 68 bp | |
| *EWSR1-FLI1* | | Thermo Fisher | Hs03024497_ft | 80 bp | |
| *IGFBP3* | | Thermo Fisher | Hs00181211_m1 | 78 bp | |
| *IGFBP5* | | Thermo Fisher | Hs00181213_m1 | 85 bp | |
| *GAPDH* | | Thermo Fisher | Hs99999905_m1 | 93 bp | |
| ***qRT-PCR primers used for ChIP assay*** | | | | | |
| **Genes** | **Sequence Forward (5′-3′)** | | **Sequence Reverse (5′-3′)** | | **Region** |
| *EZH2* | GACACGTGCTTAGAACTACGAACAG | | TTTGGCTGGCCGAGCTT | | Promoter |
| *CAV1* | GGTTCAAGAGTACATGTGCAGG | | GGGAGTAGGCTTTGTAGCTGG | | Promoter |
| *CAV1* | TTTTGCTGACCCATGCTTCTC | | GCTCTATACCCCAACATTTTCTGTG | | Enhancer |
| *AURKB* | TATGCATGTAGAGGCTCAACAAT | | TCTGGAAGTGAGGGAAGCAT | | Promoter |
| *DICER* | TCTTTTCCCCGATCTGTTGC | | TTGCTTCAGCCCAGGATTTC | | Promoter |
