# Supplementary material for: An inducible ectopic expression system of EWSR1-FLI1 as a tool for understanding Ewing sarcoma oncogenesis
Source: PLoS One. 2020 Jun 5;15(6):e0234243. doi: 10.1371/journal.pone.0234243 (PMC7274397; doi:10.1371/journal.pone.0234243)
Supplement: S3 Table — Ranked gene set: NOM p<0.05 and FDR q <0.05. (PDF) [file pone.0234243.s003.pdf]

Supplemental Table 3

Gene Set Name (pos.)	NES	NOM p-val	FDR q-val
RIGGI_EWING_SARCOMA_PROGENITOR_UP	4.758	0	0
MIYAGAWA_TARGETS_OF_EWSR1_ETS_FUSIONS_UP	4.727	0	0
ZHANG_TARGETS_OF_EWSR1_FLI1_FUSION	3.320	0	0
KINSEY_TARGETS_OF_EWSR1_FLII_FUSION_UP	3.144	0	3.29E-06
CHEN_METABOLIC_SYNDROM_NETWORK	2.923	0	6.58E-05
ONDER_CDH1_TARGETS_2_DN	2.827	0	1.78E-04
MOHANKUMAR_HOXA1_TARGETS_DN	2.772	0	2.97E-04
KRIGE_RESPONSE_TO_TOSEDOSTAT_24HR_DN	2.707	0	5.83E-04
NUYTTEN_EZH2_TARGETS_UP	2.598	3.68E-05	0.002
CHICAS_RB1_TARGETS_SENESCENT	2.586	1.39E-05	0.002
BENPORATH_SUZ12_TARGETS	2.522	3.79E-05	0.003
DELYS_THYROID_CANCER_UP	2.511	5.47E-05	0.003
DODD_NASOPHARYNGEAL_CARCINOMA_DN	2.485	1.17E-05	0.004
YAMAZAKI_TCEB3_TARGETS_UP	2.472	9.29E-05	0.004
PASINI_SUZ12_TARGETS_DN	2.418	2.14E-04	0.007
PILON_KLF1_TARGETS_DN	2.408	4.64E-05	0.007
LEE_BMP2_TARGETS_DN	2.405	1.13E-04	0.007
LIAO_METASTASIS	2.397	2.96E-04	0.007
MEISSNER_BRAIN_HCP_WITH_H3K4ME3_AND_H3K27ME3	2.389	1.35E-04	0.007
CREIGHTON_ENDOCRINE_THERAPY_RESISTANCE_3	2.351	3.80E-04	0.009
DIAZ_CHRONIC_MEYLOGENOUS_LEUKEMIA_UP	2.346	3.33E-04	0.009
FISCHER_DREAM_TARGETS	2.336	3.27E-04	0.009
ZHENG_BOUND_BY_FOXP3	2.314	5.38E-04	0.011
GRAESSMANN_APOPTOSIS_BY_DOXORUBICIN_UP	2.311	4.18E-04	0.011
BENPORATH_SOX2_TARGETS	2.274	6.48E-04	0.014
BENPORATH_NOS_TARGETS	2.267	9.14E-04	0.014
VECCHI_GASTRIC_CANCER_ADVANCED_VS_EARLY_UP	2.254	6.79E-04	0.015
FARMER_BREAST_CANCER_APOCRINE_VS_LUMINAL	2.240	0.001	0.017
WEI_MYCN_TARGETS_WITH_E_BOX	2.225	0.001	0.018
PARENT_MTOR_SIGNALING_UP	2.222	0.001	0.018
KUMAR_TARGETS_OF_MLL_AF9_FUSION	2.214	0.001	0.019
MILI_PSEUDOPODIA_HAPTOTAXIS_DN	2.208	0.001	0.019
FULCHER_INFLAMMATORY_RESPONSE_LECTIN_VS_LPS_UP	2.203	0.001	0.019
DANG_BOUND_BY_MYC	2.185	0.001	0.021
TSAI_RESPONSE_TO_IONIZING_RADIATION	2.180	0.001	0.022
LIM_MAMMARY_STEM_CELL_UP	2.167	0.002	0.023
BYSTRYKH_HEMATOPOIESIS_STEM_CELL_QTL_TRANS	2.165	0.002	0.023
PHONG_TNF_RESPONSE_NOT_VIA_P38	2.150	0.002	0.025
BASAKI_YBX1_TARGETS_DN	2.149	0.002	0.025
BOQUEST_STEM_CELL_UP	2.140	0.002	0.026
BOSCO_ALLERGEN_INDUCED_TH2_ASSOCIATED_MODULE	2.132	0.002	0.026
BENPORATH_NANOG_TARGETS	2.128	0.002	0.027
SENESE_HDAC3_TARGETS_UP	2.126	0.002	0.026
CHARAFE_BREAST_CANCER_LUMINAL_VS_BASAL_DN	2.123	0.002	0.026

KRIGE_RESPONSE_TO_TOSEDOSTAT_6HR_DN	2.113	0.002	0.028
RODWELL_AGING_KIDNEY_UP	2.112	0.003	0.027
PUJANA_BRCA1_PCC_NETWORK	2.109	0.002	0.027
NABA_CORE_MATRISOME	2.102	0.003	0.028
KEGG_PATHWAYS_IN_CANCER	2.088	0.003	0.031
KOYAMA_SEMA3B_TARGETS_UP	2.081	0.004	0.032
SMID_BREAST_CANCER_BASAL_DN	2.079	0.003	0.031
REACTOME_DEVELOPMENTAL_BIOLOGY	2.078	0.003	0.031
CREIGHTON_ENDOCRINE_THERAPY_RESISTANCE_5	2.075	0.004	0.031
SMID_BREAST_CANCER_LUMINAL_B_DN	2.073	0.003	0.031
PUJANA_ATM_PCC_NETWORK	2.068	0.003	0.031
WELCSH_BRCA1_TARGETS_UP	2.066	0.003	0.031
BLALOCK_ALZHEIMERS_DISEASE_DN	2.065	0.003	0.031
SMID_BREAST_CANCER_LUMINAL_B_UP	2.063	0.004	0.031
RUTELLA_RESPONSE_TO_CSF2RB_AND_IL4_UP	2.060	0.004	0.031
MASSARWEH_TAMOXIFEN_RESISTANCE_UP	2.052	0.004	0.032
BENPORATH_EED_TARGETS	2.045	0.003	0.033
BENPORATH_OCT4_TARGETS	2.035	0.005	0.035
KRIGE_RESPONSE_TO_TOSEDOSTAT_6HR_UP	2.033	0.004	0.035
BRUINS_UVC_RESPONSE_LATE	2.031	0.004	0.035
DOANE_RESPONSE_TO_ANDROGEN_DN	2.028	0.005	0.035
MATSUDA_NATURAL_KILLER_DIFFERENTIATION	2.024	0.005	0.035
IVANOVA_HEMATOPOIESIS_LATE_PROGENITOR	2.022	0.005	0.035
REACTOME_METABOLISM_OF_RNA	2.006	0.006	0.039
GABRIELY_MIR21_TARGETS	2.005	0.006	0.039
LIU_PROSTATE_CANCER_UP	2.004	0.005	0.038
FISCHER_DIRECT_P53_TARGETS_META_ANALYSIS	1.990	0.006	0.041
FULCHER_INFLAMMATORY_RESPONSE_LECTIN_VS_LPS_DN	1.986	0.007	0.042
DURAND_STROMA_S_UP	1.985	0.007	0.041
ZHOU_INFLAMMATORY_RESPONSE_FIMA_DN	1.982	0.007	0.042
CAIRO_HEPATOBLASTOMA_CLASSES_UP	1.982	0.007	0.041
PUJANA_CHEK2_PCC_NETWORK	1.979	0.006	0.042
THEILGAARD_NEUTROPHIL_AT_SKIN_WOUND_DN	1.974	0.007	0.042
RODRIGUES_THYROID_CARCINOMA_POORLY_DIFFERENTIATED_UP	1.973	0.007	0.042
LINDGREN_BLADDER_CANCER_CLUSTER_2B	1.971	0.007	0.042
BENPORATH_MYC_MAX_TARGETS	1.971	0.007	0.042
SPIELMAN_LYMPHOBLAST_EUROPEAN_VS_ASIAN_DN	1.956	0.008	0.045
BILD_HRAS_ONCOGENIC_SIGNATURE	1.955	0.008	0.045
MARSON_BOUND_BY_FOXP3_STIMULATED	1.953	0.007	0.045
BILD_E2F3_ONCOGENIC_SIGNATURE	1.944	0.009	0.047
STEIN_ESRRA_TARGETS	1.943	0.008	0.047
MILI_PSEUDOPODIA_CHEMOTAXIS_DN	1.942	0.008	0.046
SHETH_LIVER_CANCER_VS_TXNIP_LOSS_PAM1	1.936	0.009	0.048
BUYTAERT_PHOTODYNAMIC_THERAPY_STRESS_DN	1.935	0.009	0.047
DOUGLAS_BMI1_TARGETS_UP	1.933	0.009	0.047
BENPORATH_ES_WITH_H3K27ME3	1.930	0.007	0.048
RUIZ_TNC_TARGETS_UP	1.928	0.010	0.048

VERHAAK_AML_WITH_NPM1_MUTATED_DN	1.922	0.010	0.049
GRAESSMANN_RESPONSE_TO_MC_AND_DOXORUBICIN_UP	1.922	0.010	0.049

Gene Set Name (neg.)	***NES***	***NOM p-val***	***FDR q-val***
**RIGGI_EWING_SARCOMA_PROGENITOR_DN**	-2.782	2.86E-05	4.52E-04
PLASARI_TGFB1_TARGETS_10HR_DN	-2.029	0.004	0.052
**MIYAGAWA_TARGETS_OF_EWSR1_ETS_FUSIONS_DN**	-2.025	0.004	0.036
